# From trainees to trainers to instructors: Sustainably building a national capacity in bioinformatics training

**DOI:** 10.1371/journal.pcbi.1006923

**Published:** 2019-06-27

**Authors:** Annette McGrath, Katherine Champ, Catherine A. Shang, Ellen van Dam, Cath Brooksbank, Sarah L. Morgan

**Affiliations:** 1 Data61, CSIRO, Brisbane, Queensland, Australia; 2 Bioplatforms Australia, Sydney, New South Wales, Australia; 3 EMBL-EBI, Wellcome Genome Campus, Hinxton, Cambridge, United Kingdom; University of Toronto, CANADA

## Abstract

Demand for training life scientists in bioinformatics skills led to the development of a train-the-trainer collaboration between the European Molecular Biology Laboratory–European Bioinformatics Institute (EMBL-EBI) and 2 Australian organisations, Bioplatforms Australia and Commonwealth Scientific and Industrial Research Organisation (CSIRO) in 2012. The goal of the collaboration was to establish a group of trained instructors who could develop and deliver short bioinformatics courses nationally. A train-the-trainer course introduces instructors to aspects of andragogy and evidence-based learning principles to help them better design, develop, and deliver high-quality training. Since then, both the number of trainers in the network and the course portfolio have grown. Best practises have been developed and shared between the Australian cohort and EMBL-EBI to address common challenges in bioinformatics training. The Australian trainer cohort undertook a train-the-trainer instructor course, again with EMBL-EBI, and subsequently successfully delivered train-the-trainer courses to interested bioinformatics trainers within Australia. We conclude that a train-the-trainer approach can help build national capacity and maintain a critical mass of trained instructors.

This is a *PLOS Computational Biology* Education paper.

## Introduction

In recent years, there has been an inexorable increase in the growth of data production in the life sciences, which has led to a shift in the practise of life science research itself. Applied bioinformatics skills are now essential to the successful analysis of data from high-throughput ‘omics instrumentation. Despite the demonstrated need for bioinformatics skills, bioinformatics seldom occupies a core part of undergraduate life science degree courses. Yet productive life scientists need some degree of familiarity with bioinformatics concepts, tools, resources, and approaches to design and execute their experiments appropriately. This leads many researchers, at all career stages, to seek out point-of-need courses: when the need arises, they seek training in that particular topic to enable them to perform their immediate data analysis tasks.

Bioinformatics itself is a rapidly evolving multidisciplinary science that requires biological, statistical, and computational expertise. Technological advances in instrumentation often necessitate the development of new tools and methods. This creates an ongoing need for bioinformatics skills development for bioinformaticians and experimental biologists alike, who need to constantly adapt and learn new skills. The level of demand for bioinformatics training has been increasing in recent years [1,2,3].

In 2012, despite the high demand for bioinformatics training, there was a shortage of courses and trainers in Australia. Surveys undertaken within institutions confirmed the need for training in a wide variety of topics, driven by the availability of increasing volumes of data [4]. To address this need, a train-the-trainer (TtT) programme was implemented through a collaboration between Bioplatforms Australia (Bioplatforms), the Commonwealth Scientific and Industrial Research Organisation (CSIRO), and the European Molecular Biology Laboratory–European Bioinformatics Institute (EMBL-EBI) training team. This programme aimed to sustainably increase the number of local workshops by leveraging existing EMBL-EBI material and expanding the Australian volunteer training base.

The TtT programme instructed active bioinformatics researchers in aspects of educational theory and assisted them with the design and subsequent delivery of a course in Australia, using EMBL-EBI training material as a foundation. This work has been previously described [4].

Since this initial TtT workshop, over 30 bioinformatics training workshops have been delivered by the cohort of TtT attendees to 930 researchers in 13 institutions in 8 cities around Australia (Table 1). Over 20 Australian bioinformaticians have participated in the TtT programme, a number of whom undertook the first EMBL-EBI/ELIXIR TtT instructor course in 2016. In turn, these trainers have organised and hosted Australian-based TtT workshops, further building the national bioinformatics training capacity.

**Table 1:** The number of trainees taught in courses hosted across Australia by the Australian TtT cohort.

Workshop information	Count
**Bioplatforms/CSIRO/EMBL-EBI bioinformatics workshops hosted**	31
**Bioinformatics training workshop attendees**	930
**Number of bioinformatics training workshop applicants**	1,342
**Bioplatforms/CSIRO bioinformaticians that have attended a TtT course**	22
**Number of institutions visited by TtT attendees to deliver bioinformatics workshops**	13
**Number of different subject matter workshops delivered utilising training material developed through the TtT programme**	7

Abbreviations: CSIRO, Commonwealth Scientific and Industrial Research Organisation; EBI, European Bioinformatics Institute; EMBL, European Molecular Biology Laboratory; TtT, train the trainer

This paper describes the components of the TtT programme and how the collaboration expanded beyond the initial Introduction to Next-Generation Sequencing (NGS) workshop in 2012. The overall success of this programme has led to outcomes beyond the original scope of this programme. In the intervening period, there has been shared learning by all partners in this collaboration. This paper discusses what we have learned and shared with each other and demonstrates how national bioinformatics training capacity was sustainably increased using this model.

## Establishing the TtT model

**Fig 1:**
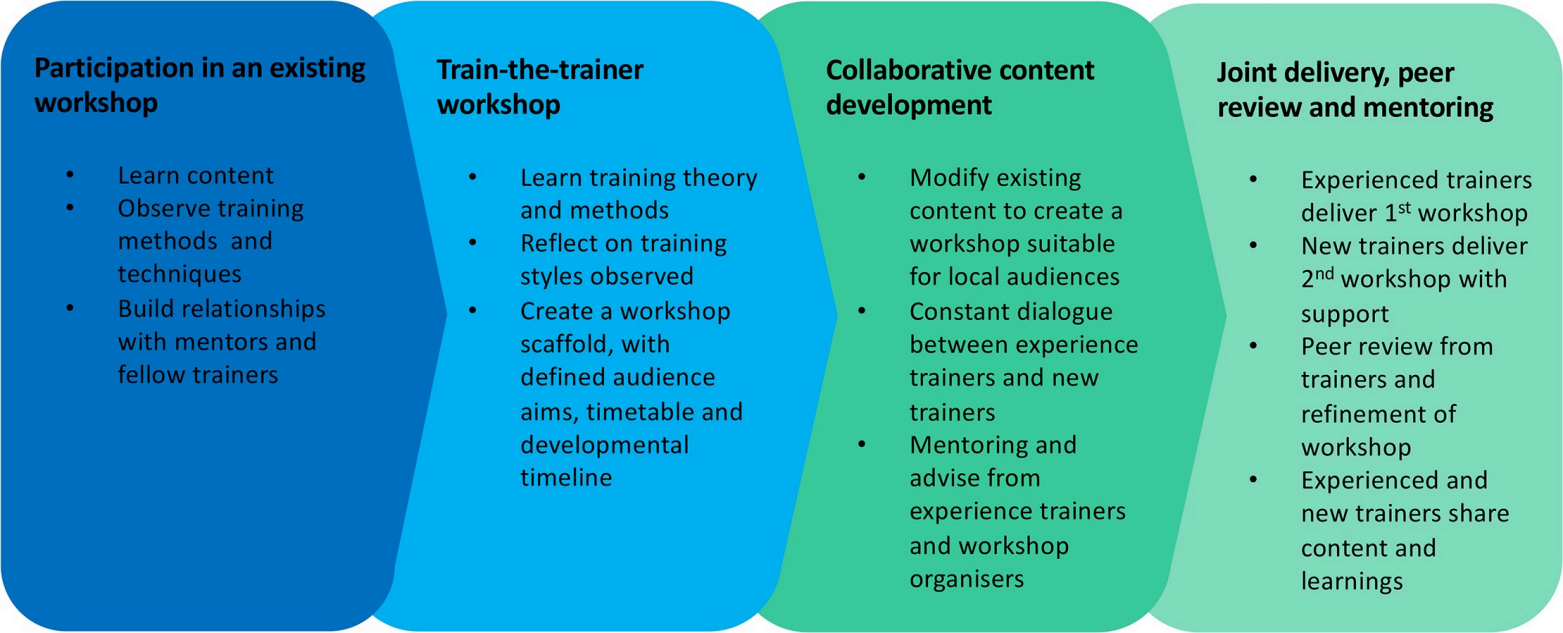
Components of the EMBL-EBI, Bioplatforms Australia, and CSIRO train-the-trainer model. CSIRO, Commonwealth Scientific and Industrial Research Organisation; EBI, European Bioinformatics Institute; EMBL, European Molecular Biology Laboratory.

The TtT model used by this programme incorporated several components: observation and participation in an established workshop; instruction on training theories and methodologies; collaborative content development and delivery; and, importantly, coaching and mentoring from experienced trainers with peer-to-peer support (Fig 1). With these elements at its core, the TtT programme allowed new trainers to develop and deliver world-class content whilst being supported and mentored by expert instructors.

### Participation in an established workshop

In March 2012, a group of 8 Australian bioinformatics researchers with an interest in bioinformatics training were selected from Bioplatforms Australia and CSIRO facilities to participate in the TtT programme. The participants were geographically dispersed with varying research interests but were all experienced in the analysis of NGS data. The group attended an established 3-day introductory course at EMBL-EBI on NGS data analysis, where they participated actively and observed experienced trainers delivering the course.

### TtT workshop

The cohort then attended a 2-day TtT workshop, designed by EMBL-EBI, which offered practical guidance on course development and delivery based on educational principles. Many in the Australian group were first-time trainers or had no prior exposure to training theories. As such, it was important to introduce a range of communication and training methods, as well as concepts around different learning styles and preferences. During the TtT workshop, the new trainers reflected on and discussed their experiences from the EMBL-EBI NGS workshop from an attendee’s perspective. The group discussed the elements that contributed to good or bad training sessions, drawing attention to their own learning preferences and how these could influence their training styles.

The TtT workshop also addressed practicalities around course design, including defining the target audience and learning objectives, accommodating different learning styles, programme design, and gathering and assessing feedback from attendees. The workshop highlighted how these tools and methodologies could help focus the development and refinement of training content. These lessons were put into practise on day 2 of the course, when the Australian cohort, with EMBL-EBI’s input, developed an outline for an NGS workshop to be delivered upon their return home.

The Australian cohort selected core modules, or subsets of topics, from the EMBL-EBI NGS workshop that, from their experience, met the needs of researchers in their own institutes. They then developed a set of learning objectives for the new course, identifying infrastructure requirements and data sources. By the end of the TtT workshop, the Australian cohort and EMBL-EBI faculty had developed an outline for a 2-day Introduction to NGS workshop, using the EMBL-EBI content as a foundation.

### Collaborative course development

To maintain momentum, a fixed time frame of 4 months after the EMBL-EBI workshop was set for the first delivery of the new Australian course. The course outline and material were refined and prepared with the aid of regular Skype meetings between EMBL-EBI trainers and the Australian cohort. The course outline was expanded to document which EMBL-EBI material (presentations, datasets, tutorial exercises) would be reused to meet the learning objectives of the course, to what extent they would be adapted, and also to develop any new material as needed.

As the project involved multiple contributors in different geographical locations, Google Docs was used to share course material and documentation, enabling multiple users to contribute and track changes. With the success of the workshop series and the growth of the volunteer trainer base, in subsequent years a robust reproducible framework for developing, maintaining, and delivering material using GitHub was developed and adopted by the group [5].

As the workshop would be run in several locations across the country, a mode of delivery that would minimise reliance on information technology (IT) staff also needed to be found. As earlier described, a cloud-based solution was chosen [6].

### Joint delivery, mentoring, and review

This first Australian course was delivered in July 2012 in Sydney and Melbourne, with the majority of the content being delivered by the EMBL-EBI faculty and the Australian cohort assisting as trainers or as helpers. After these first 2 courses, the Australian cohort reviewed feedback from the EMBL-EBI faculty and the trainees. They reflected on their own experience of having participated as training assistants and determined how to further adapt the material for the local audience. This led to alterations to the course before it was run next, later in 2012. Most notably, this included the addition of an introductory command line session. Continual evaluation of the feedback from later workshops led to the course growing from the initial 2-day workshop to a 3-day workshop.

The TtT programme was fundamental to the successful development and delivery of the Australian Introduction to NGS course. The programme taught practical aspects of course design and delivery whilst giving the new trainers access to established training materials. It introduced formal training methodologies and allowed trainers to put their learning into practise within a short time frame, with expert support. The additional postcourse coaching and mentoring also reinforced the concepts that they had learned, providing them with the confidence to deliver the material themselves.

## Growing the portfolio and the network

Feedback from the Introduction to NGS workshops indicated that there was considerable demand for workshops in other bioinformatics analysis topics. However, with only 8 volunteer trainers, the network was underresourced to fulfil this demand. To deliver a bioinformatics workshop, trainers were required to take time from their research to travel and teach, attend preworkshop teleconferences to discuss required updates, and assist with the maintenance of the material and training platforms. Therefore, to meet the community's needs, the Bioplatforms–CSIRO training portfolio and network was expanded through TtT programmes at EMBL-EBI and The Genome Analysis Centre (TGAC; now the Earlham Institute) (Table 2).

**Table 2:** Courses attended at European institutions to grow the Australian course portfolio and trainer network.

Course	Institution	Date	Length	AU trainers attending	Length of AU course	Date AU course first run	Number of original faculty attended
Introduction to Metagenomics	EMBL-EBI	September 2013	3 days	8	2 days	February 2014	3
Cancer Genomics	EMBL-EBI	July 2015	5 days	7	3 days	November 2015	2
De Novo Genome Assembly	TGAC	July 2015	2 days	8	2.5 days	November 2015	1

Abbreviations: AU, Australian; EBI, European Bioinformatics Institute; EMBL, European Molecular Biology Laboratory; TGAC, The Genome Analysis Centre

As with the initial NGS workshop, subsequent courses were adapted from existing workshops to meet Australian researchers’ needs and a broader audience. As such, introductory content was often expanded, and complex or specific exercises were removed. The trainers also added datasets relevant to the Australian research context and materials from their own training portfolio.

Following these 4 TtT courses, the course portfolio had expanded to 7 (S1 Table) in response to community demand. More focussed and in-depth courses on popular topics such as RNA-Seq were requested. Trainers added new material to the TtT-developed material to create these. On other occasions, shorter introductory courses were requested. The modular nature of the courses meant that a new course from existing content could be readily created. Additionally, the trainer network had grown to over 20 individuals (with some attending more than 1 TtT course), thereby creating a cohesive, engaged, enthusiastic, and suitably trained volunteer training workforce that also supported and coached each other in their training delivery.

## Developing and sharing best practises

As the Bioplatforms/CSIRO training portfolio and network grew, the EMBL-EBI and Australian teams continued to engage in practical, technological, and evaluation strategies to support both the training programme and the trainers. EMBL-EBI shared further in-house processes for trainee selection and expectation management and impact evaluation, including gathering long-term feedback. Similarly, the Australian cohort shared their own training experiences, feedback, and observations with EMBL-EBI so that they in turn could assess the impact of their TtT courses and inject new ideas into current course offerings.

### Common issues

It became apparent that there are common issues associated with delivering bioinformatics training courses to diverse audiences in different contexts. These included inspiring, enthusing, and keeping participants engaged; gaining useful feedback on whether learning is taking place; and defining methods that actually work for training delivery. In this collaboration, we shared efforts to meet and overcome these challenges through development and solidification of best practises. These issues are common among trainers worldwide. Working with EMBL-EBI raised awareness among the trainers from Australia of international initiatives to address common challenges in bioinformatics training. This led them to participate in and directly influence international bioinformatics training initiatives including the Global Organisation for Bioinformatics Learning, Education, and Training (GOBLET [7]), the International Society on Computational Biology Education committee, ELIXIR, and Big Data to Knowledge (BD2K).

### What the Australian cohort learned

In addition to drawing on EMBL-EBI’s expertise and training support, the Australian team adapted or developed practises required to run their workshops in a local context, which were constantly reviewed to ensure that they met the needs of the trainers and trainees. Ultimately, these also influenced how EMBL-EBI delivers its own training. For example, some tutorial example datasets used in the Australian workshops were shared with EMBL-EBI and subsequently used in EMBL-EBI workshops.

The road show format of the workshops and the distributed trainer network necessitated the development of innovative solutions for sharing course material, developing it collaboratively, and delivering courses with minimal overhead for host institutions [5,6]. The Australian trainers were committed to the creation of a community-focussed initiative and an open-access policy for infrastructure and content. They were early adopters of GitHub for shared maintenance of course material. All material was made publicly available on GitHub for reuse.

Bioinformatics training courses require sufficient compute resources, successful installation of multiple tools, and availability of reference data. A cloud-based training platform was developed that served a number of purposes: it addressed the need to minimise preparation for IT administrators at host institutions, provided a consistent software environment for all trainees, provided a storage solution for reference and test datasets, and enabled course participants to access a virtual machine (VM) on the cloud after the course, thereby enabling them to reinforce their learning.

Furthermore, early experiences with the Australian NGS course indicated that there was demand for a stand-alone RNA-Seq course using several components of the NGS course (e.g., alignment and RNA-Seq). Modularising the course into discrete topics, such as alignment and RNA-Seq, meant that it was possible to design bespoke courses from the content that had already been developed by the team. Each module was further broken down into 4 components necessary to rerun a workshop reproducibly: tools, data, manual, and presentation material. This modularisation allowed a ‘mix-and-match’ approach, allowing trainers to select short or longer courses depending on local training needs [6].

Bioinformatics training courses are not static and need updating with technological advances. With some courses that were run less frequently (e.g., Introduction to Metagenomics), EMBL-EBI trainers shared updated materials and their latest example datasets before each new iteration of the course.

### What has EMBL-EBI learned?

The development of the TtT programme with the Australian bioinformatics community was EMBL-EBI’s first move towards developing a formal TtT programme for both an external and an internal audience. The programme has developed significantly since its initiation in 2012 on the basis of feedback from the organisers and participants of the initial courses, allowing greater emphasis to be placed on certain elements of the training and how best to deliver to, and inspire confidence in, the new trainers.

Since this collaboration started, TtT courses have been delivered with a number of communities who were looking to further develop training capacity, enabling a focus on specific subjects as the start point for that development. For example, the Capacity Building for Bioinformatics in Latin America (CABANA) project (www.cabana.online) is building bioinformatics training capacity throughout Latin America, using a modification of the TtT model initially developed for Australia.

Whilst EMBL-EBI was basing its own training on the principles taught in the TtT activities with Australia, it had no formal mechanism for developing its own trainers when the collaboration began. In 2014, EMBL-EBI launched its internal TtT programme. This is broadly similar to that developed through the Australian collaboration, with some additions. For example, after participating in a TtT course, new trainers are allocated a mentor to provide ongoing support as the trainer develops his or her training skills. Efforts made to identify training opportunities soon after a new trainer has completed the TtT course enable new trainers to put what they have learned into practise.

The principles laid down through this programme have also fed into the development of the ELIXIR TtT initiative funded by the ELIXIR EXCELERATE project, as highlighted in recent publications [8,9].

## Coming full circle: TtT instructor course

In May 2016, a community survey by the Australian Bioinformatics and Computational Biology Society (ABACBS) education subcommittee found a strong interest in participating in a TtT workshop as part of the ABACBS/GOBLET Best Practices in Bioinformatics Training workshop, which was being held in conjunction with the GOBLET Annual General Meeting (AGM) 2016 and the ABACBS 2016 national conference (https://www.abacbs.org/goblet/workshops/).

A TtT instructor (TtTI) course was initiated, developed, and delivered by EMBL-EBI, with members of the Bioplatforms/CSIRO network of trainers as instructors in training. Eight members of the training network volunteered to participate in a TtTI course. Participants were coached remotely to deliver existing TtT material through a series of 6 webinar sessions in September and October 2016. This training material was based on the ELIXIR EXCELERATE TtT programme [8], which in turn had been inspired by this group’s earlier work with EMBL-EBI [4] and Software Carpentry/Data Carpentry (https://carpentries.org/). The 6 sessions covered (1) an introductory session explaining the approach to the TtTI course and course structure and addressing trainee questions; (2) the elements of good versus bad training and trainers; (3) reviewing how people learn; (4) defining learning objectives and using them to design sessions and courses; (5) assessment and gathering feedback; and (6) a final wrap-up session to address any questions or clarifications arising from the course.

To maximise the coaching time, materials were shared beforehand so that the instructors in training had an opportunity to review them and prepare questions. Additional background material was provided for greater understanding of the theoretical background behind the presentation material. In the coaching sessions, the presentation material was reviewed, and activities for instructors to facilitate discussion of these concepts to trainees were explored. Approximately 30 minutes of each session was dedicated to questions and answers, which gave instructors in training the opportunity to delve deeper into the material with the coach.

The support and experience of EMBL-EBI was of considerable benefit to the instructors in training, who drew on this knowledge during the formal coaching sessions. Prior to the TtT course delivery day and having reviewed the training material, an additional coaching question-and-answer session was held to boost trainers’ confidence and to clarify any further questions from the instructor trainers. Whilst the instructors in training still felt some trepidation, they felt well prepared to deliver the TtT workshop by the end of the TtTI course. Although the TtTI course contains elements of educational theory, its main focus is on the practical elements of teaching people in a workshop setting. All the instructors in training had lots of experience, and that was as valuable as the basic theory. In essence, the TtT course focusses on individuals making practical changes to the way they do things.

## Training others

The GOBLET/ELIXIR TtT course was held on Nov 4, 2016, with 15 trainees, the majority of whom were already experienced trainers. As the focus of the TtT course is on sharing experiences and ideas, a group of this size easily allows these discussions to happen and be managed by the instructors.

There was no selection process for the course, but all participants were sent a survey prior to the course to detail their experience of training to date and their expectations in attending. This enabled the instructors to gain a better overview of the prospective attendees and enable them to prepare to meet certain expectations. This exercise also helped the trainees prepare, getting them to think about the experiences and ideas they could share and what kind of support they were looking for within their own training practise.

The course was delivered by 4 of the 8 TtT instructor trainees, with 1 trainer being responsible for each of the 4 main topics in the course.

Directly after the course, an anonymous feedback survey was conducted about the organisation and content of the course, whether the trainees felt that they would use the material in their future training activities, open questions about best and worst parts of the course, and any requests for additional course content.

Despite the fact that many attendees were active trainers themselves, the feedback was overwhelmingly positive, with all attendees indicating that they had gained something from the course. One hundred percent of attendees stated that they would use what they had learned in future courses and also indicated that they would recommend the course to others. Attendees in particular enjoyed the interactive and collaborative format of the course and learning from the experience of others.

As a number of course attendees had some training experience and may naturally already be undertaking some of the recommendations in the TtT, the course content provided them with an opportunity to reflect on their own training practise and incorporate tips and tricks from other participants. Despite their experience, attendees commented on how the TtT course had made them look at how they train in a very different light. Knowing that they need to structure their lessons and exercises in different ways to cater to individuals’ different learning styles and considering how writing learning objectives would help them to communicate what a lesson or course will cover resonated strongly with them.

Following the success of this course, a TtT workshop was again delivered at the ABACBS national conference in November 2017 to 5 attendees. To date, a total of 20 new Australian trainers have been trained by the Bioplatforms/CSIRO instructors, enabling further growth of the national cohort of trained bioinformatics trainers, bringing the total of Australian trainers who have been part of this programme, at EMBL-EBI or in Australia, to 42.

## Trainer benefits from the TtTI course

**Fig 2:**
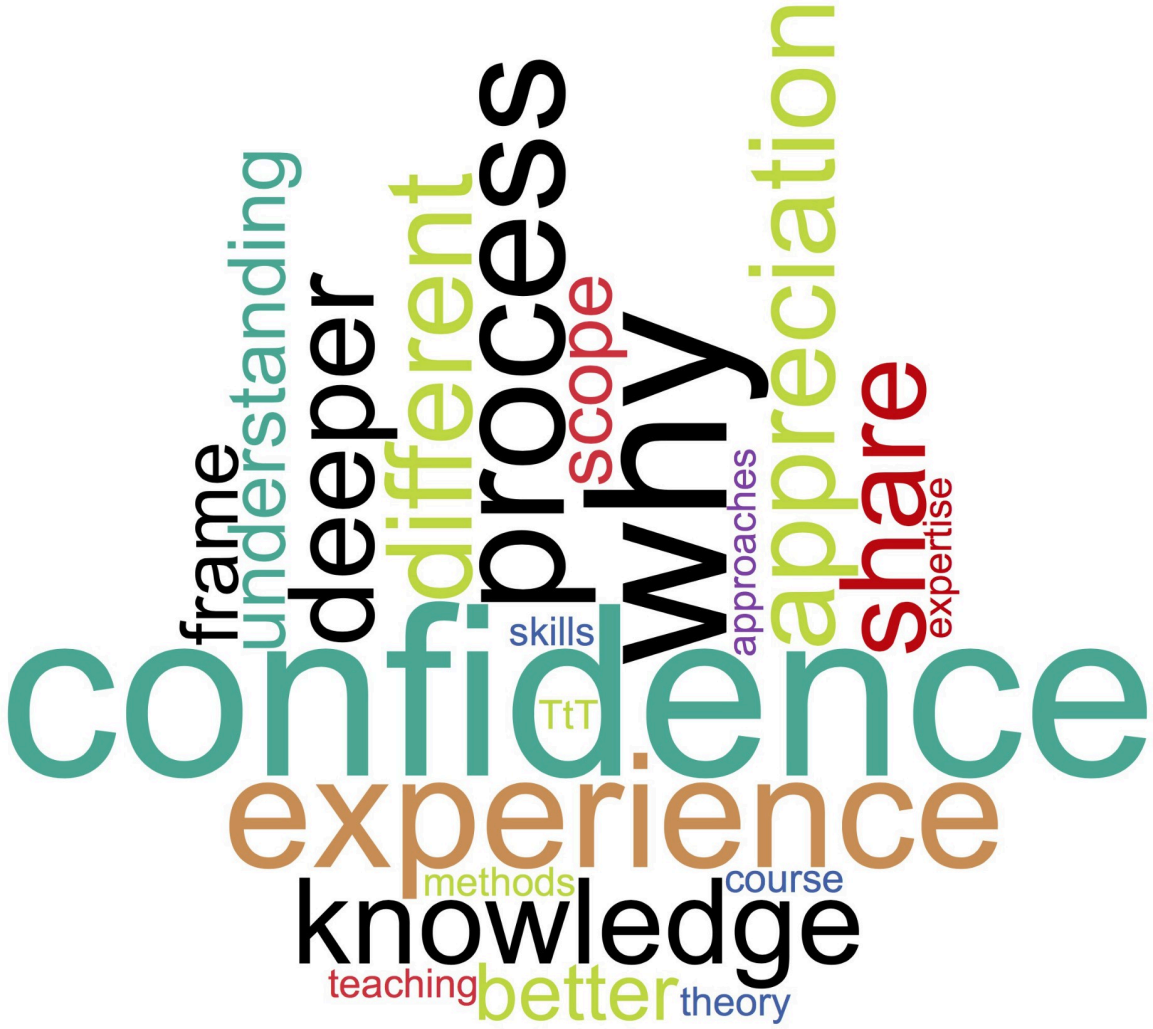
Feedback from instructors in training following the TtTI course, describing how it helped them to train others.

Feedback on the benefit of the TtTI course from the new instructors who delivered the course indicated that the course provided them with a useful refresher on the TtT course content, a greater knowledge and appreciation of the course topics, a framework by which to share their experiences, and the confidence to deliver a TtT course (Fig 2). None of them had otherwise considered doing so, despite their extensive training experience. The TtTI course provided a formalised way of passing on what they learned from their own experiences to others. For example, hearing the trainees describe examples of good and bad training and trainers provided an excellent avenue for trainers to share some of their experiences. This experience also allowed them to synthesise what attendees were outlining into higher-level concepts and provide linkages between topics, broadening trainees’ thinking about those topics.

Trainers also noted that when attending a TtT course as trainees, they focussed on the ‘how’: how to apply what they were learning to the course they would design and teach. They felt that the TtTI course focussed more on the ‘why’: why we teach these topics in a TtT course, why it matters to trainees, and ultimately why it makes a difference to the attendees of the courses they will deliver. This was similar to their own experiences teaching technical topics to bioscientists, such as quality control of NGS data. They know this topic well but don’t necessarily have either the need or the time to share all of that knowledge with attendees for the attendees to learn about this. However, without this deep subject matter knowledge, they felt that they would fall short on teaching the topic and that consequently the TtTI course provided that knowledge to them. In this context, the TtTI course provides attendees with the best way to impart their skills to attendees.

There was also a greater appreciation among the new instructors of what the original TtT courses they undertook gave them. For example, the instructors remembered not particularly enjoying having to write intended learning outcomes for the course that they would create in their TtT course and found it a very difficult task. However, with course design, teaching, and TtTI experience behind them, they had a newfound appreciation for why this exercise is essential and now appreciate that it is fundamental in framing the scope and indeed the limits of the course based on the audience that you are aiming your course at.

## Conclusion

The TtT model, successfully developed in 2012 between an Australian trainer cohort and EMBL-EBI, has been expanded to increase the course portfolio and the network of trainers in Australia who have undertaken the TtT programme. The TtT model takes a group of bioinformaticians with subject matter expertise, enthusiasm, and ability to learn and trains them in aspects of andragogy and good course design, followed by subsequent mentoring and coaching and an opportunity to put what they have learned into practise shortly after completion of the TtT course. Twenty-two Australian trainers have been trained in this way by EMBL-EBI, and almost 1,000 trainees in Australia have attended a variety of courses, using EMBL-EBI training material as their foundation, delivered by this group over a 5-year period.

On participating in a TtT workshop in Europe, the Australian trainers agreed to volunteer to deliver a minimum number of workshops nationally for the Bioplatforms/CSIRO programme. All 22 trainers met that requirement. In many cases, these trainers also participate in developing and delivering these or other workshops within their own institutions or local communities, as supported by their workplace. The TtTI initiative enabled another 20 Australian trainers, not associated with either Bioplatforms or CSIRO, to undertake a TtT workshop that they could then apply to their own training. Those trainers are, to our knowledge, still involved in training at their own institutions or in local communities.

The TtT training and mentoring provided this cohort with confidence and assurance and also a streamlined guide to developing and updating material. Without the opportunity to attend an EMBL-EBI course and subsequent trainer training, it is highly unlikely that they would have come together to develop a course such as this without the focus that this opportunity gave them.

**Fig 3:**
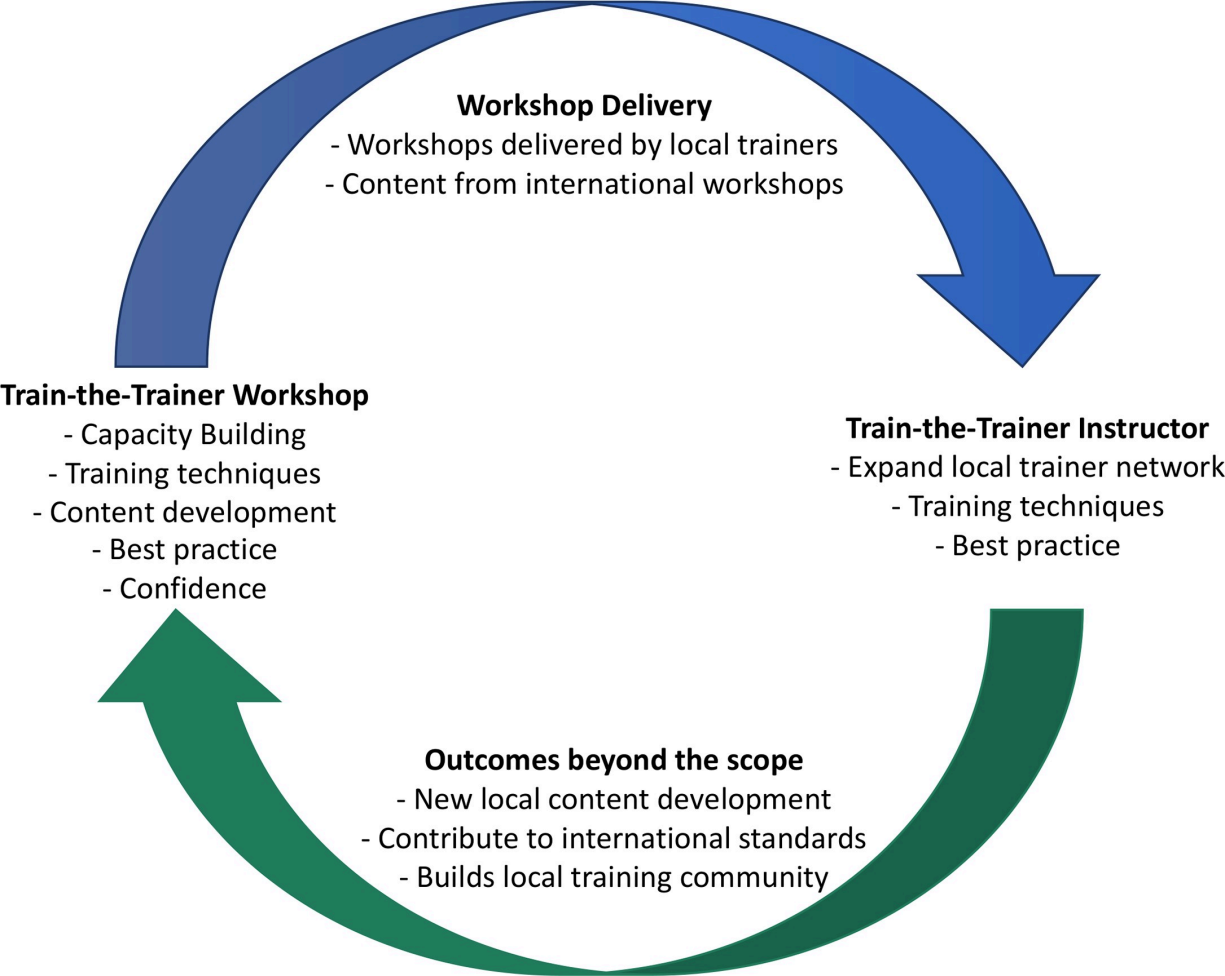
Outcomes beyond the scope of the initial TtT and workshop delivery have been achieved.

Outcomes beyond the scope of the initial collaboration have been achieved (Fig 3). What began as training a single cohort of bioinformaticians to become bioinformatics trainers has progressed to the point where trainers in Australia can maintain their own ‘pipeline’ of new bioinformatics trainers because a subset of them are now competent TtT instructors. Their familiarity with the TtT programme and experiences gained since then provided them with many examples to share with new trainers, and the course gave them the necessary tools and confidence to continue to the next step.

The TtTI initiative enabled a new group of Australian trainers to benefit from a TtT course and in doing so enabled the Bioplatforms/CSIRO trainers to reinforce their own learning and reflect on their own practise. This allowed not only independent capacity building but also local community building in the Australian training network.

With a TtT workshop delivered at the national bioinformatics conference in 2 consecutive years, the total number of Australian trainers who have benefited from this programme is 42 over 5 years, substantially increasing the national bioinformatics training capacity. Having a larger pool of trained trainers increases sustainability of training capability nationwide and minimises impacts from trainers moving on to new roles and being unavailable to train.

Identifying common issues faced by trainers in delivering training to diverse audiences helped the collaboration become more of a shared best practises effort. Each group shared their solutions to particular challenges they faced in evaluation of learning, identification of suitable test datasets, training delivery, sharing content with other trainers, content maintenance and updating, and trainee engagement. This led to involvement with global professional bodies for bioinformatics training and engagement with other training consortia meant that both groups could contribute to initiatives to address these challenges.

The demand for bioinformatics and data science training in the life sciences continues [1,2,3], and initiatives such as this demonstrate how a cohort of trained bioinformatics trainers can be established using a TtT programme. Future plans to sustain and maintain the training programme will build on the current model of operation and expand to new themes, maintaining quality and currency through collaboration and coordination via continued engagement with EMBL-EBI and other leading international bioinformatics groups.

## Supporting information

S1 Table Workshop portfolio. (DOCX)
