# Supplementary material for: From trainees to trainers to instructors: Sustainably building a national capacity in bioinformatics training
Source: PLoS Comput Biol. 2019 Jun 27;15(6):e1006923. doi: 10.1371/journal.pcbi.1006923 (PMC6597034; doi:10.1371/journal.pcbi.1006923)
Supplement: S1 Table — (DOCX) [file pcbi.1006923.s001.docx]

| **Workshop Name** | **Content Information** |
| --- | --- |
| 2-day Introduction to Next Generation Sequencing (NGS) workshop | Original Introduction to Next Generation Sequencing (NGS) workshop: technology introduction, quality control, alignment, ChIP-Seq, RNASeq,  bacterial de novo assembly |
| 3-day Introduction to NGS workshop | Original workshop plus an introduction to command-line |
| 1/2-Day Introduction to NGS, quality and alignment | Technology introduction, command-line, quality control and alignment |
| 1-Day Introduction to RNA-Seq Data Analysis | Technology introduction, command-line, quality control, alignment and RNA-Seq Data Analysis |
| 2-Day Introduction to RNA-Seq and ChIP-Seq Data Analysis | Technology introduction, command-line, quality control, alignment, ChIP-Seq and RNA-Seq Data Analysis |
| 2-Day Introduction to Metagenomics workshop | Technology introduction, command-line, quality control and alignment modules from NGS workshops |
| 3-day Cancer Genomics Workshop | Technology introduction, command-line, quality control and alignment modules from NGS workshops (1-day) plus cancer genomics specific content (2 days): Single nucleotide variant detection, CNV detection, structural variants, Somatic mutation analysis, visualizing variants |
| 2.5 day de novo assembly workshop * | Technology introduction, command-line, quality control, alignment and basic de novo modules from NGS workshops (1 day) plus (eukaryotic) de novo assembly content |

S1 Table1: Course portfolio
